# Acute Neuromuscular Responses and Interlimb Asymmetry Following 5 km and Half-Marathon Running in Recreational Runners

**DOI:** 10.3390/biology15191682

**Published:** 2026-09-22

**Authors:** Danijela Kuna, Petar Šušnjara, Klara Findrik

**Affiliations:** 1 Faculty of Kinesiology Osijek, Josip Juraj Strossmayer University of Osijek, 31000 Osijek, Croatia; psusnjara@kifos.hr (P.Š.); kfindrik@kifos.hr (K.F.); 2 Faculty of Kinesiology, University of Zagreb, 10000 Zagreb, Croatia

**Keywords:** tensiomyography, vastus medialis, muscle contractile properties, maximal voluntary isometric strength, interlimb asymmetry, endurance running

## Abstract

Acute neuromuscular responses to running may vary according to exercise duration and physiological demands. This study compared changes in vastus medialis contractile properties, quadriceps maximal voluntary isometric contraction (MVIC), and interlimb asymmetry following 5 km and half-marathon running trials. Twenty-eight male recreational runners (36.96 ± 11.35 years) completed both trials 10 days apart. Maximal radial displacement (Dm), contraction time (Tc), and contraction velocity (Vc) were assessed using tensiomyography, while quadriceps MVIC was assessed using isometric dynamometry before and after each trial. Absolute interlimb asymmetry and its direction were also analysed. The unadjusted analysis showed a greater pre-to-post increase in Dm following the half-marathon; however, the effect of the running trial on post-running Dm was not significant after adjustment for baseline Dm. Tc and MVIC decreased after both trials, whereas Vc increased, with no significant running trial × time interactions for these outcomes. TMG- and MVIC-derived absolute interlimb asymmetry remained unchanged. Acute post-running responses differed according to the neuromuscular characteristic assessed rather than consistently according to running distance. Changes in contractile properties and maximal force-generating capacity were not accompanied by a systematic increase in the magnitude of between-leg differences.

## Simple Summary

Running over different distances challenges the body in varying ways, but whether shorter and longer efforts lead to distinct immediate changes in muscle function remains unclear. This study examined 28 male recreational runners before and after a 5-kilometre run and a half-marathon. We assessed how a thigh muscle responded to electrical stimulation, how much force the quadriceps muscles could produce during maximal effort, and whether the difference between the right and left legs changed after running. The quadriceps produced less maximal force after both distances, while some characteristics of muscle contraction also changed. The size of the difference between the two legs did not increase after either run. A larger change in muscle movement was initially observed after the half-marathon, but the difference between the two running trials was no longer significant after accounting for pre-run values. These findings show that different aspects of muscle function can respond differently immediately after running and may help improve understanding of acute muscle responses in recreational runners.

## 1. Introduction

The 5 km and half-marathon distances are highly popular among recreational runners [1]. In their weekly training programmes, runners most commonly combine shorter, higher-intensity training sessions with longer continuous training sessions aimed at developing endurance [2]. This pattern of physical loading involves frequent contact with the ground and repeated exposure to high forces, requiring the neuromuscular system of the lower extremities to continually adapt motor unit recruitment and interlimb load distribution [2,3]. Under such conditions, the quadriceps, hamstrings, and lower-leg muscles are exposed to acute changes in force-generating capacity and contractile properties, which may manifest as a reduction in maximal isometric strength and alterations in the mechanical response of the muscles after running [3,4,5]. These responses involve distinct features of neuromuscular function and may vary according to the characteristics of the running task.

Although the cardiovascular and metabolic responses to running at different durations and intensities have been relatively well described, a growing body of research emphasises that key performance-related adaptations and potential injury-related mechanisms occur specifically at the neuromuscular and biomechanical levels, particularly with the onset of fatigue [3,6]. The accumulation of fatigue during half-marathon running may reduce the neuromuscular system’s ability to generate force effectively, which is reflected in changes in the biomechanical parameters of running. These may include a prolonged stance phase, reduced peak vertical ground reaction forces, and changes in impulse, thereby altering the way in which the lower extremities absorb and transmit loads during running [3,7]. Such changes suggest that running-related loading affects not only movement patterns but also the functional state of the lower-extremity muscles. Characterising the acute neuromuscular response to running therefore requires consideration of both muscle contractile behaviour and force-generating capacity. In this context, tensiomyography (TMG) enables a non-invasive assessment of the muscle’s contractile response to electrical stimulation without requiring any additional voluntary effort from the participant. The most commonly analysed parameters include maximal radial muscle displacement (Dm) and contraction time (Tc). Dm reflects the magnitude of radial muscle displacement and is commonly interpreted in relation to the muscle’s mechanical and contractile state, whereas Tc describes the temporal component of the electrically evoked response. Previous studies have shown that TMG parameters may be sensitive to fatigue-induced changes and useful for assessing the acute muscular response following various forms of lower-extremity loading, including running-related loading [5,8,9]. However, the interpretation of changes in individual TMG parameters may depend on the muscle examined, the characteristics of the exercise protocol, the timing of post-exercise assessment, and the standardisation of the measurement procedure. Their responses should therefore be interpreted within the specific context in which the method is applied.

The vastus medialis muscle is particularly relevant to the present study. As part of the quadriceps, the vastus medialis contributes to knee extension and patellar stability. Changes in its contractile properties following running may indicate an acute muscular response that is not necessarily fully apparent through the measurement of maximal force alone. TMG assessment of the vastus medialis provides muscle-specific information on its electrically evoked mechanical response rather than a direct measure of voluntary force production.

Dynamometric assessment of maximal voluntary isometric contraction (MVIC) of the quadriceps enables the evaluation of voluntary maximal isometric force-generating capacity. Unlike TMG, which characterises the muscle’s contractile response to stimulation, MVIC reflects the functional outcome of the combined neural and peripheral factors involved in force production. Previous research has shown that half-marathon running may lead to a reduction in maximal voluntary isometric force of the knee extensors, supporting the use of MVIC for assessing acute changes in force-generating capacity following prolonged running [10]. TMG and isometric dynamometry thus characterise different neuromuscular outcomes: an electrically evoked muscle response and voluntary force-generating capacity, respectively. Assessing both outcomes in the same runners allows their respective acute response patterns to be compared within the same experimental context. In addition to the measured values of neuromuscular parameters, increasing attention has been paid to bilateral asymmetry in strength and biomechanical variables [11,12,13]. Bilateral asymmetry during running largely depends on running speed and the runners’ training status. Competitive runners tend to exhibit a linear reduction in asymmetry as the running speed increases, whereas recreational runners display a non-linear pattern, achieving the greatest degree of symmetry at their preferred running speed [14]. A systematic review of the literature indicates that measures of functional, morphological, kinematic, and kinetic lower-extremity asymmetry are generally negatively associated or not associated with middle- and long-distance running performance, although these findings are highly heterogeneous [11]. Conversely, some studies suggest that symmetry may even improve under certain fatigue conditions. Among recreational runners, a reduction in the asymmetry angle of peak vertical ground reaction force has been observed at the end of a half-marathon, indicating the use of active compensatory strategies to maintain balance in response to fatigue [7]. Furthermore, longitudinal studies show that biomechanical asymmetries in runners do not change significantly from season to season; however, specific reductions in asymmetry, such as propulsive impulse asymmetry, are significantly associated with better personal race records [15]. The interpretation of interlimb asymmetry may therefore benefit from considering both its magnitude and direction, as these provide information on the extent of the difference and the limb in which the higher value occurs.

Despite growing evidence on neuromuscular responses to endurance running, direct within-subject comparisons of acute responses following shorter and longer running distances remain limited. It is not yet clear whether vastus medialis contractile properties, quadriceps maximal voluntary isometric force, and interlimb asymmetry show different pre-to-post response patterns following 5 km and half-marathon running. Previous studies have also rarely considered both the magnitude and direction of interlimb asymmetry alongside the original neuromuscular variables in the same participants. Comparing these outcomes within the same recreational runners may help clarify whether acute neuromuscular responses differ consistently with running distance or instead depend on the specific characteristic assessed.

Therefore, this study aimed to compare acute neuromuscular responses to 5 km and half-marathon running in recreational runners. Specifically, we examined pre-to-post changes in vastus medialis Dm, Tc, and Vc, quadriceps MVIC, and the magnitude and direction of interlimb asymmetry.

## 2. Materials and Methods

### 2.1. Participants

The sample consisted of 28 male recreational runners with a mean age of 36.96 ± 11.35 years. An a priori sample-size calculation was performed using G*Power (version 3.1.9.7), assuming an effect size of dz = 0.60, an alpha level of 0.05, and statistical power (1 − β) of 0.80. The analysis indicated a required sample size of 25 participants. All participants had been actively involved in recreational road running for at least five years. Eligibility criteria included being older than 18 years and having no musculoskeletal injury during the previous six months that could affect running performance or participation in the testing procedures. All participants were informed about the objectives and procedures of the study and provided written informed consent before testing. The study was conducted in accordance with the Declaration of Helsinki, and the research protocol was approved by the Ethics Committee of the Faculty of Kinesiology Osijek (approval no. 029-01/25-01/05; 17 July 2025).

### 2.2. Experimental Design

The study was conducted in November 2025 using a within-subject repeated-measures design. The 5 km trial was performed first, followed by the half-marathon 10 days later, a sequence predefined to minimise the possibility that residual fatigue from the longer trial could affect subsequent 5 km performance and to allow adequate recovery between trials. During the inter-trial period, participants were instructed to avoid exhaustive or high-intensity training, particularly in the 48 h preceding the second trial. Training load and physical activity were not objectively monitored. Both trials were conducted under simulated competitive conditions. Participants ran in small groups of no more than three runners. The groups were formed according to comparable previous performances over the respective distances to ensure similar running dynamics within each group. Running pace was individually selected, and participants were instructed to maintain a high but sustainable effort according to their current fitness level and previous experience over the respective distances. Participants were also asked to report their best 5 km and half-marathon completion times achieved during the preceding 12 months. These self-reported performances were used to contextualise the relative performance level of each running trial. Relative performance was calculated as the best reported completion time for the corresponding distance divided by the trial completion time × 100.

At the beginning of the study, anthropometric measurements were obtained and body composition was assessed. Before each running trial, participants completed a standardised 10 min dynamic warm-up according to a predefined protocol. Following the warm-up, baseline assessments of vastus medialis contractile properties using TMG and quadriceps maximal voluntary isometric contraction (MVIC) using isometric dynamometry were performed. Post-running TMG assessment commenced approximately 2 min after the completion of each running trial and required approximately 7 min to assess both legs. The same leg-assessment order was maintained across all TMG measurements. Quadriceps MVIC assessment followed immediately after the completion of the TMG measurements. During the immediate post-running assessment period, participants were permitted to take small sips of water if they felt the need to drink.

### 2.3. Measurements

#### 2.3.1. Body Composition and Heart Rate Monitoring

Body mass index and body composition data were obtained using a segmental body composition analyser (Tanita BC-601, Tanita Corporation, Tokyo, Japan). The variables included in the analysis were body mass index (kg·m^−2^), body fat percentage (%), and muscle mass percentage (%). Measurements were performed under standardised conditions, with participants barefoot and wearing light clothing.

Heart rate (HR) was continuously monitored during each running trial using a chest-strap heart rate monitor (Polar H10, Polar Electro Oy, Kempele, Finland) synchronised with the participant’s smartwatch. Mean and peak heart rate values were retained for analysis as indicators of the cardiovascular response during the two running trials.

#### 2.3.2. Tensiomyographic Assessment

Tensiomyography (TMG) was used to assess the contractile properties of the vastus medialis muscle bilaterally using a TMG device (TMG-BMC, Ljubljana, Slovenia) equipped with a digital displacement sensor (GK40, Panoptik, Ljubljana, Slovenia). The sensor was positioned perpendicular to the skin surface over the thickest part of the muscle belly. Two self-adhesive electrodes (5 cm × 5 cm; Compex Medical SA, Ecublens, Switzerland) were positioned symmetrically on either side of the sensor and aligned with the direction of the muscle fibres. Electrode and sensor placement followed the manufacturer’s recommendations. The sensor and electrode positions were marked on the skin with a skin marker to ensure consistent placement between the pre- and post-run assessments.

Participants were assessed in a supine position, with the knee supported at 40° of flexion using the manufacturer’s wedge cushion. Measurements were performed under static conditions while the muscle was fully relaxed, and radial displacement of the muscle belly was recorded during electrically evoked contractions. Single electrical stimuli of 1 ms duration were applied, starting at 20 mA and increasing in 10 mA increments until no further increase in maximal radial displacement (Dm) was observed. A 10 s interval was maintained between successive stimuli. The analysed TMG parameters were maximal radial displacement (Dm), defined as the maximum amplitude of radial muscle displacement, and contraction time (Tc), defined as the time interval between 10% and 90% of the maximal contraction response. Contraction velocity between 10% and 90% of maximal radial displacement (Vc) was additionally calculated as 0.8 × Dm/Tc. Values calculated in mm/ms were multiplied by 1000 and expressed in mm/s. All TMG assessments were performed in the same laboratory under similar ambient conditions (approximately 21–23 °C).

#### 2.3.3. Isometric Dynamometry

Quadriceps maximal voluntary isometric contraction (MVIC) was assessed bilaterally using an isometric knee dynamometer (S2P, Science to Practice Ltd., Ljubljana, Slovenia). Measurements were performed with the participant in a seated position, with an angle of 85° between the trunk and thigh and the knee flexed to 60°. The trunk and the pelvis were stabilised with straps to minimise compensatory movements. Leg dominance was determined by self-report, with participants asked to identify the leg they considered stronger and preferentially used for tasks such as kicking a ball. The dominant leg was assessed first, followed by the non-dominant leg. Before the recorded trials, participants performed one familiarisation contraction to become accustomed to the testing procedure. The protocol consisted of three maximal isometric knee-extension contractions for each leg, each lasting three seconds, with a 30 s rest interval between attempts. The highest force value recorded across the three attempts for each leg was retained for analysis and expressed in newtons (N).

#### 2.3.4. Interlimb Asymmetry Calculations

The TMG software 3.6 (TMG-BMC d.o.o., Ljubljana, Slovenia) generated a lateral symmetry score (LS) for the vastus medialis based on bilateral delay time (Td), contraction time (Tc), sustain time (Ts), and maximal radial displacement (Dm), according to the standard TMG algorithm [16]:

$$LS=100\times \left[0.1\frac{min\left(Td_{R},Td_{L}\right)}{max\left(Td_{R},Td_{L}\right)}+0.6\frac{min\left(Tc_{R},Tc_{L}\right)}{max\left(Tc_{R},Tc_{L}\right)}+0.1\frac{min\left(Ts_{R},Ts_{L}\right)}{max\left(Ts_{R},Ts_{L}\right)}+0.2\frac{min\left(Dm_{R},Dm_{L}\right)}{max\left(Dm_{R},Dm_{L}\right)}\right]$$

TMG-derived absolute interlimb asymmetry was calculated as:

TMG absolute asymmetry (%) = 100 − *LS*

A value of 0% indicated complete symmetry, whereas higher values indicated greater between-leg differences.

Directional interlimb asymmetry was calculated separately for Dm, Tc, and MVIC using the following equation:

$$Directional\;asymmetry\;(\%)\;=\;\frac{\left(R\;-\;L\right)}{\max\left(R,\;L\right)}\;\times \;100.$$

Positive values indicated a higher value in the right leg, whereas negative values indicated a higher value in the left leg. A value of 0 indicated identical right- and left-leg values, for which no asymmetry direction was assigned.

Absolute MVIC-derived interlimb asymmetry was calculated as:

Absolute MVIC asymmetry (%) = $\frac{\left|R-L\right|}{max(R,\;L)}$ × 100, with higher values indicating greater between-leg differences.

### 2.4. Statistical Analysis

Statistical analyses were conducted using JASP, version 2.6.44.0. Descriptive statistics were calculated for all variables and included the mean (M), standard deviation (SD), minimum (Min), and maximum (Max). For directional asymmetry, frequencies (n) and relative percentages (%) were additionally reported according to the limb in which the higher value was observed.

Distributional characteristics were assessed using the Shapiro–Wilk test (W), skewness and kurtosis with their standard errors, and visual inspection of histograms and Q–Q plots. For paired-samples comparisons, normality was assessed for the paired differences. Numerical normality diagnostics for absolute interlimb asymmetry are provided in Supplementary Table S1, with corresponding histograms and Q–Q plots presented in Figures S1 and S2. The differences in running performance and cardiovascular response between the 5 km and half-marathon trials were analysed using paired-samples *t*-tests. Running pace, relative performance, mean heart rate, and peak heart rate were compared between the two trials. Effect sizes were expressed as Cohen’s d. Because the distribution of paired differences in running pace deviated from normality, this comparison was additionally verified using the Wilcoxon signed-rank test.

Changes in Dm, Tc, Vc, and MVIC were analysed using repeated-measures analyses of variance. Each model included three within-subject factors: running trial, time, and leg. The running trial factor included the 5 km and half-marathon trials, the time factor included the pre- and post-running measurements, and the leg factor included the right and left legs. The main effects of all three factors, as well as their two-way and three-way interactions, were examined. The results were reported as F-values (F), degrees of freedom (df), *p*-values, and partial eta squared (ηp^2^). When significant interaction effects were identified, simple main effects analyses were conducted. Because baseline Dm differed between the two trial days, an additional baseline-adjusted linear mixed-effects model was fitted with the post-running Dm as the dependent variable and the baseline Dm as a covariate. Running trial, leg, and the running trial × leg interaction were included as fixed effects, with participant included as a random intercept. Denominator degrees of freedom were estimated using the Satterthwaite approximation. To examine possible trial-day differences in baseline MVIC, baseline values were additionally analysed using a repeated-measures ANOVA with running trial and leg as within-subject factors.

Absolute interlimb asymmetry in the TMG and MVIC parameters was analysed using separate repeated-measures analyses of variance with two within-subject factors: running trial and time. Because several absolute-asymmetry distributions deviated from normality, the corresponding running trial, time, and running trial × time contrasts were additionally examined using Wilcoxon signed-rank tests as sensitivity analyses. For these analyses, the time contrast compared mean Pre and Post values across trials, the running-trial contrast compared mean 5 km and half-marathon values across time points, and the interaction contrast compared the Pre-to-Post change between the two running trials. Rank-biserial correlation (r_rb) was reported as the effect size for these sensitivity analyses. Participant-level changes in asymmetry direction from pre- to post-running were additionally examined for Dm, Tc, and MVIC by identifying whether the limb with the higher value changed between measurements. Participants with identical right- and left-leg values at either measurement were excluded from the corresponding switching denominator. The level of statistical significance was set at *p* < 0.05 for all analyses.

## 3. Results

### 3.1. Participant Characteristics, Running Performance, and Cardiovascular Response

Table 1 presents descriptive indicators of participant characteristics, running performance, and cardiovascular responses during the 5 km and half-marathon running trials. The mean running pace was faster during the 5 km trial than during the half-marathon trial, corresponding to approximately 4:05 min/km and 4:36 min/km, respectively. Relative performance was similar across the two trials, averaging 94.90 ± 3.39% for the 5 km trial and 94.36 ± 3.93% for the half-marathon trial. Higher mean and peak heart rate values were also recorded during the 5 km trial.

Paired-samples comparisons showed that the 5 km trial was completed at a significantly faster pace than the half-marathon trial, t(27) = −8.407, *p* < 0.001, Cohen’s d = −1.589. This finding was also confirmed by the Wilcoxon signed-rank test (*p* < 0.001). Relative performance did not differ between the 5 km and half-marathon trials, t(27) = 0.595, *p* = 0.557, Cohen’s d = 0.112. Mean heart rate was significantly higher during the 5 km trial, t(27) = 3.737, *p* < 0.001, Cohen’s d = 0.706, as was peak heart rate, t(27) = 2.809, *p* = 0.009, Cohen’s d = 0.531.

### 3.2. Neuromuscular Outcomes Assessed by Tensiomyography and Isometric Dynamometry

Table 2 presents the TMG and MVIC parameters before and after the 5 km and half-marathon running trials. Descriptively, Dm and Vc values increased, whereas Tc and MVIC values decreased following both trials. Absolute interlimb asymmetry values showed relatively small numerical differences across measurements.

Table 3 summarises the repeated-measures ANOVA results for Dm, Tc, Vc, and MVIC. The unadjusted repeated-measures ANOVA showed a significant running trial × time interaction for Dm, F(1, 27) = 5.756, *p* = 0.024, ηp^2^ = 0.176. Dm did not change significantly after the 5 km trial (*p* = 0.193) but increased after the half-marathon trial (*p* < 0.001). However, baseline Dm was higher before the 5 km trial than before the half-marathon trial (*p* = 0.001), and after adjustment for baseline Dm, the effect of running trial on post-running Dm was not significant, F(1, 85.17) = 1.415, *p* = 0.238 (Table S3). Tc and MVIC decreased from Pre to Post across both running trials, with significant main effects of time for Tc, F(1, 27) = 7.187, *p* = 0.012, ηp^2^ = 0.210, and MVIC, F(1, 27) = 24.563, *p* < 0.001, ηp^2^ = 0.476. Vc increased after running, F(1, 27) = 27.991, *p* < 0.001, ηp^2^ = 0.509. For Tc, Vc, and MVIC, no significant running trial × time interactions were observed, with no statistical evidence that the pre-to-post changes differed between the two running trials. Baseline MVIC did not differ significantly between the two trial days, F(1, 27) = 0.182, *p* = 0.673, ηp^2^ = 0.007. A significant main effect of leg was observed, F(1, 27) = 11.285, *p* = 0.002, ηp^2^ = 0.295, whereas the running trial × leg interaction was not significant, F(1, 27) = 0.161, *p* = 0.691, ηp^2^ = 0.006.

To illustrate individual variability in the pre-to-post Dm response, Figure 1 presents participant-level trajectories for the 5 km and half-marathon running trials.

### 3.3. Interlimb Asymmetry

The repeated-measures ANOVA results for absolute interlimb asymmetry are presented in Table 4. Neither TMG- nor MVIC-derived absolute interlimb asymmetry showed significant main effects of running trial or time, and no significant running trial × time interactions were observed. These findings were unchanged in the Wilcoxon signed-rank sensitivity analyses (Table S2).

Because absolute asymmetry indices indicate the magnitude of interlimb differences but not their direction, an additional descriptive analysis of directional asymmetry was performed. As shown in Table 5, higher MVIC values were more frequently observed in the right leg across all measurement conditions. Higher Dm values were more frequently observed in the left leg, except after the 5 km trial, when the frequencies were equal between the legs.

Higher Tc values were more frequently observed in the right leg at each measurement point, although two participants had equal right- and left-leg values at both half-marathon measurements. The predominant direction of the interlimb difference therefore differed across Dm, Tc, and MVIC.

Participant-level changes in asymmetry direction from Pre to Post were observed for all three outcomes. The side with the higher value changed in 35.7% and 33.3% of participants for Dm, 39.3% and 36.0% for Tc, and 42.9% and 28.6% for MVIC following the 5 km and half-marathon trials, respectively.

## 4. Discussion

### 4.1. Main Findings

This study examined the acute neuromuscular responses of recreational runners to 5 km and half-marathon running trials, which differed in distance, running pace, and cardiovascular response. The unadjusted analysis showed a significant running trial × time interaction for maximal radial displacement (Dm), with Dm increasing after the half-marathon but not after the 5 km trial. However, baseline Dm was lower before the half-marathon, and the effect of running trial on post-running Dm was not significant after adjustment for baseline Dm.

Tc and MVIC decreased, whereas Vc increased after both running trials, with no significant running trial × time interactions for these outcomes. Furthermore, neither TMG- nor MVIC-derived absolute interlimb asymmetry changed significantly after running or differed between the two trials. Overall, the findings indicate parameter-specific acute neuromuscular responses, while interpretation of Dm was influenced by between-day baseline variation and absolute interlimb asymmetry showed no systematic change.

### 4.2. Tensiomyographic Responses of the Vastus Medialis Muscle

Although Dm increased significantly after the half-marathon but not after the 5 km trial in the unadjusted analysis, baseline Dm was already lower before the half-marathon. After baseline adjustment, no significant running-trial effect on post-running Dm remained, limiting attribution of the larger unadjusted change to running distance. Higher Dm values are commonly interpreted as reflecting lower muscle stiffness or greater tissue compliance, although the interpretation of Dm may also depend on the muscle and functional context [17,18,19]. The relationship between Dm and muscle stiffness is not strictly linear, however, and Dm should not be treated as a direct measure of overall muscle mechanical properties [17,18]. The observed changes may therefore reflect an altered mechanical or contractile state of the muscle, without identifying the underlying mechanism.

This interpretation is supported by Giovanelli et al. [20], who observed an increase in rectus femoris Dm after a mountain marathon and associated this response with reduced muscle stiffness and altered responsiveness to electrical stimulation. Gutiérrez-Vargas et al. [8] similarly reported an increase in rectus femoris Dm following a marathon performed under hot and humid conditions. García-Manso et al. [21] also identified muscle-specific TMG changes in the rectus femoris and biceps femoris after an ultra-endurance triathlon. These studies provide context for the direction of the Dm change observed after the half-marathon trial, although the baseline-adjusted analysis in the present study does not support interpreting this response as a clear effect of running distance.

TMG responses following fatiguing exercise are not uniform across exercise protocols or muscles. Yeom et al. [22] reported that reductions in Dm and Tc are frequently observed following acute muscle fatigue. Piqueras-Sanchiz et al. [23] also observed reductions in Dm in the vastus medialis and vastus lateralis following a strength-exercise protocol designed to induce peripheral fatigue. Such variability can be related to exercise modality and duration, the muscle examined, participant characteristics, and the timing of the post-exercise assessment. The Dm findings should therefore be interpreted within the specific context of the vastus medialis assessment and the observed between-day baseline variability, rather than as evidence of a universal or distance-dependent TMG response to running-induced fatigue. For Tc, a significant main effect of time was observed without a running trial × time interaction, with shorter contraction times after both running trials. This reduction should not be interpreted as evidence against fatigue. TMG-derived Tc is not a unidirectional marker of fatigue and also reflects the immediate contractile state of the muscle. Abazović et al. [24] reported shorter TMG-derived Tc in both the vastus medialis and vastus lateralis during the first two minutes following a post-activation potentiation stimulus. Martín-San Agustín et al. [9] found limited responsiveness of Tc in the vastus medialis and rectus femoris following a quadriceps fatigue protocol. Given that TMG assessment in the present study commenced approximately 2 min after running, the shorter Tc could represent the combined influence of fatigue and transient post-activation changes during the early recovery period rather than fatigue alone. Vc increased after both running trials, with no significant running trial × time interaction. Because Vc was derived from Dm and Tc, this change captures their combined post-running behaviour rather than an independent physiological response. Previous studies likewise show that individual TMG parameters can respond differently to the same exercise stimulus. Rojas-Valverde et al. [25] found that TMG parameters, including Dm and Tc, changed during a simulated duathlon, although the pattern depended on the exercise phase and parameter assessed. Taken together, the present findings support interpreting Dm, Tc, and Vc as related but distinct characteristics of the electrically evoked contractile response rather than as uniform indicators of a single fatigue process.

### 4.3. Quadriceps Maximal Voluntary Isometric Strength

In addition to the changes in TMG parameters, the reduction in quadriceps MVIC demonstrates an acute decline in maximal isometric force-generating capacity after running. MVIC decreased significantly after both trials, and the magnitude of the reduction did not differ significantly between them. The absence of a running trial × time interaction provides no evidence that the half-marathon produced a greater acute loss of force than the 5 km trial. Baseline MVIC also did not differ significantly between trial days, providing no statistical evidence of a systematic between-day difference in maximal isometric force before running. The two trials nevertheless imposed different combinations of running demands. The 5 km trial was completed at a faster pace and elicited higher mean and peak heart rate values, reflecting a higher cardiovascular response over a shorter period. Conversely, the half-marathon involved a substantially longer exercise duration and a greater number of repeated loading cycles. Despite these differences, relative performance was comparable between trials. The similar post-running decline in MVIC could therefore arise from different combinations of running pace, cardiovascular strain, exercise duration, and accumulated mechanical loading. This interpretation is supported by our previous findings in recreational runners, in whom quadriceps maximal voluntary isometric strength decreased after a 5 km run [26]. Boccia et al. [10,27] also reported impaired knee-extensor force-generating capacity following a half-marathon. In one of these studies, the ability to develop force rapidly was also impaired after the half-marathon [27], showing that running-related neuromuscular changes can extend beyond maximal force alone. In a subsequent study, Boccia et al. [10] reported a reduction in knee-extensor maximal voluntary isometric force following a half-marathon. The inclusion of electrically evoked contractions allowed the authors to distinguish more clearly between the central and peripheral components of fatigue. The present study, however, assessed changes in MVIC without additional measures of voluntary activation or electrically evoked force responses. The observed reduction in MVIC demonstrates an acute loss of maximal isometric strength but does not allow the relative contributions of central and peripheral mechanisms to be identified. Reductions in knee-extensor force have also been reported under other running conditions. Márquez et al. [28] observed lower maximal voluntary contraction and evoked contractile responses after a progressive running test to exhaustion, whereas Coratella et al. [29] reported reduced MVIC following downhill running. Together, these findings show that a reduction in maximal isometric force can occur across markedly different running protocols. Similar MVIC changes therefore do not imply equivalent physiological or mechanical demands. Brownstein et al. [30] likewise reported comparable reductions in maximal voluntary contraction despite differing neural and contractile contributions to fatigue. Because voluntary activation and evoked force were not assessed in the present study, MVIC should be interpreted here as a functional measure of post-running force loss rather than as evidence of a specific fatigue mechanism.

In addition to the significant main effect of time, a significant main effect of leg was observed, with MVIC values generally higher in the right leg than in the left leg. However, the absence of significant interactions involving leg indicates that this difference remained relatively consistent across the measurement conditions and that the pre-to-post pattern did not differ significantly between the legs.

### 4.4. Magnitude and Direction of Interlimb Asymmetry

Although Dm, Tc, and MVIC changed after running, neither TMG- nor MVIC-derived absolute interlimb asymmetry showed significant main effects of running trial or time, and no significant running trial × time interactions were observed. Thus, post-running changes in the individual neuromuscular variables were not accompanied by a systematic increase in the magnitude of between-leg differences. The absence of a significant change in absolute interlimb asymmetry can be considered in relation to the cyclical and alternating nature of running. Both legs repeatedly contribute to propulsion, support, and load absorption through broadly comparable movement patterns. Such bilateral loading could limit the development of a systematic increase in interlimb asymmetry after either running trial. This explanation remains tentative because mechanical loading of each leg was not measured directly. This interpretation is consistent with the findings of Heil et al. [31], who reported that fatigue-related changes in asymmetry depend on the exercise protocol, test, and parameter assessed. Parkinson et al. [32] further emphasised that conclusions regarding asymmetry depend on the calculation method and the specific characteristic being measured. Interlimb asymmetry should be considered in relation to both the task and the variable from which it is derived. The additional descriptive analysis of directional asymmetry showed that the leg displaying the higher value differed according to the parameter assessed. Higher MVIC values were more frequently observed in the right leg, which is consistent with the significant main effect of leg for maximal isometric force. In contrast, higher Dm values were more frequently observed in the left leg, particularly after the half-marathon trial. Higher Tc values were more frequently observed in the right leg at each measurement point, although equal right- and left-leg values occurred in two participants at both half-marathon measurements.

The side displaying the higher value was not necessarily maintained from Pre to Post within individual participants. Changes in asymmetry direction occurred in 35.7% and 33.3% of participants for Dm, 39.3% and 36.0% for Tc, and 42.9% and 28.6% for MVIC following the 5 km and half-marathon trials, respectively. These descriptive shifts occurred despite the absence of significant changes in absolute asymmetry, showing that stability in asymmetry magnitude does not imply stability in its direction. The predominance of higher MVIC values in the right leg was not mirrored by Dm. This difference is physiologically plausible because MVIC represents voluntary force production, whereas Dm quantifies radial muscle displacement in response to electrical stimulation. Consequently, the leg with the higher value for one outcome need not show the higher value for another. The participant-level changes in direction further demonstrate that an unchanged group-level asymmetry magnitude does not imply that the same limb consistently retains the higher value. Considering both magnitude and direction therefore provides a more complete description of interlimb relationships. Future studies should examine whether longer endurance events produce different changes in asymmetry magnitude or direction and should report both components when characterising interlimb responses.

### 4.5. Relationship Between Electrically Evoked and Voluntary Neuromuscular Responses

TMG and MVIC showed different post-running response profiles. Tensiomyography characterised the electrically evoked contractile response of the vastus medialis, whereas MVIC represented voluntary quadriceps force-generating capacity. Tc and Vc changed after both trials, interpretation of Dm was influenced by between-day baseline variation, and MVIC declined irrespective of running distance. These findings indicate that the pattern of acute post-running change differed across the neuromuscular characteristics assessed. Post-running changes in Dm, Tc, Vc, and MVIC were not accompanied by systematic changes in absolute interlimb asymmetry, further distinguishing changes in individual neuromuscular variables from changes in between-leg differences.

### 4.6. Limitations and Future Directions

Several limitations should be considered. Although the final sample exceeded the number required by the a priori power analysis, it included only male recreational runners and covered a broad range of ages and performance levels. The findings cannot be directly generalised to female runners, elite athletes, or more homogeneous performance groups. Maximal heart rate was not independently assessed, so heart-rate responses could not be expressed relative to individual HRmax and were used only to characterise the cardiovascular response during the two running trials. The fixed trial order, with all participants completing the 5 km trial before the half-marathon, does not allow potential order effects to be fully excluded. This sequence was predefined to minimise possible residual fatigue from the longer half-marathon on the subsequent 5 km performance, but a randomised or counterbalanced design would have provided stronger control of order-related influences. Baseline Dm values were also significantly lower before the half-marathon trial despite the same measurement procedures being applied on both testing days. The source of this baseline discrepancy cannot be determined from the present design. The effect of running trial on post-running Dm was not significant after adjustment for baseline Dm. Although participants were instructed to avoid exhaustive or high-intensity training between trials, particularly during the 48 h preceding the second trial, training load and physical activity were not objectively monitored. Therefore, differences in activity during the inter-trial period cannot be excluded as a contributor to between-day variability. TMG assessment was limited to the vastus medialis muscle, and the findings cannot therefore be generalised to the entire quadriceps or other lower-extremity muscles. Including additional quadriceps muscles and the hamstrings would have provided a broader representation of lower-limb contractile responses to running. In addition, muscle and skin temperature, hydration status, and exercise-related fluid shifts were not directly quantified and could therefore not be considered when interpreting the TMG-derived responses. Consequently, the relative contributions of central and peripheral fatigue to the reduction in MVIC could not be determined, so the observed reduction represents a functional loss of maximal force without identifying its underlying mechanism. TMG was assessed before MVIC during post-exercise testing, with TMG commencing approximately 2 min after running and MVIC performed immediately after completion of the bilateral TMG measurements. Thus, the two outcomes were obtained at different points of early recovery rather than simultaneously.

Measurements were performed only immediately before and after the 5 km and half-marathon trials. Consequently, the duration and recovery trajectory of the observed changes remain unknown, and the results cannot be directly extrapolated to marathon or ultra-endurance events. In addition, the trials were conducted under simulated competitive conditions, and motivational, tactical, and environmental influences could have differed from those experienced during official races.

Future studies should include both female and male runners, use randomised or counterbalanced trial orders, assess additional quadriceps muscles and the hamstrings, and include repeated measurements throughout recovery. Objective monitoring of training between trials and the inclusion of voluntary activation and electrically evoked force responses would further improve the interpretation of post-running neuromuscular changes.

## 5. Conclusions

The present study showed that 5 km and half-marathon running trials produced parameter-specific acute neuromuscular changes in male recreational runners. The unadjusted analysis showed a greater pre-to-post Dm increase following the half-marathon. However, the effect of running trial on post-running Dm was not significant after adjustment for baseline Dm. Tc and quadriceps MVIC decreased following both trials, whereas Vc increased. Neither TMG- nor MVIC-derived absolute interlimb asymmetry changed significantly. These results indicate that acute post-running responses differed according to the neuromuscular characteristic assessed rather than consistently according to running distance. The absence of systematic changes in absolute interlimb asymmetry further indicates that acute alterations in muscle contractile properties and maximal force-generating capacity were not accompanied by an increase in the magnitude of between-leg differences.

## Figures and Tables

**Figure 1:**
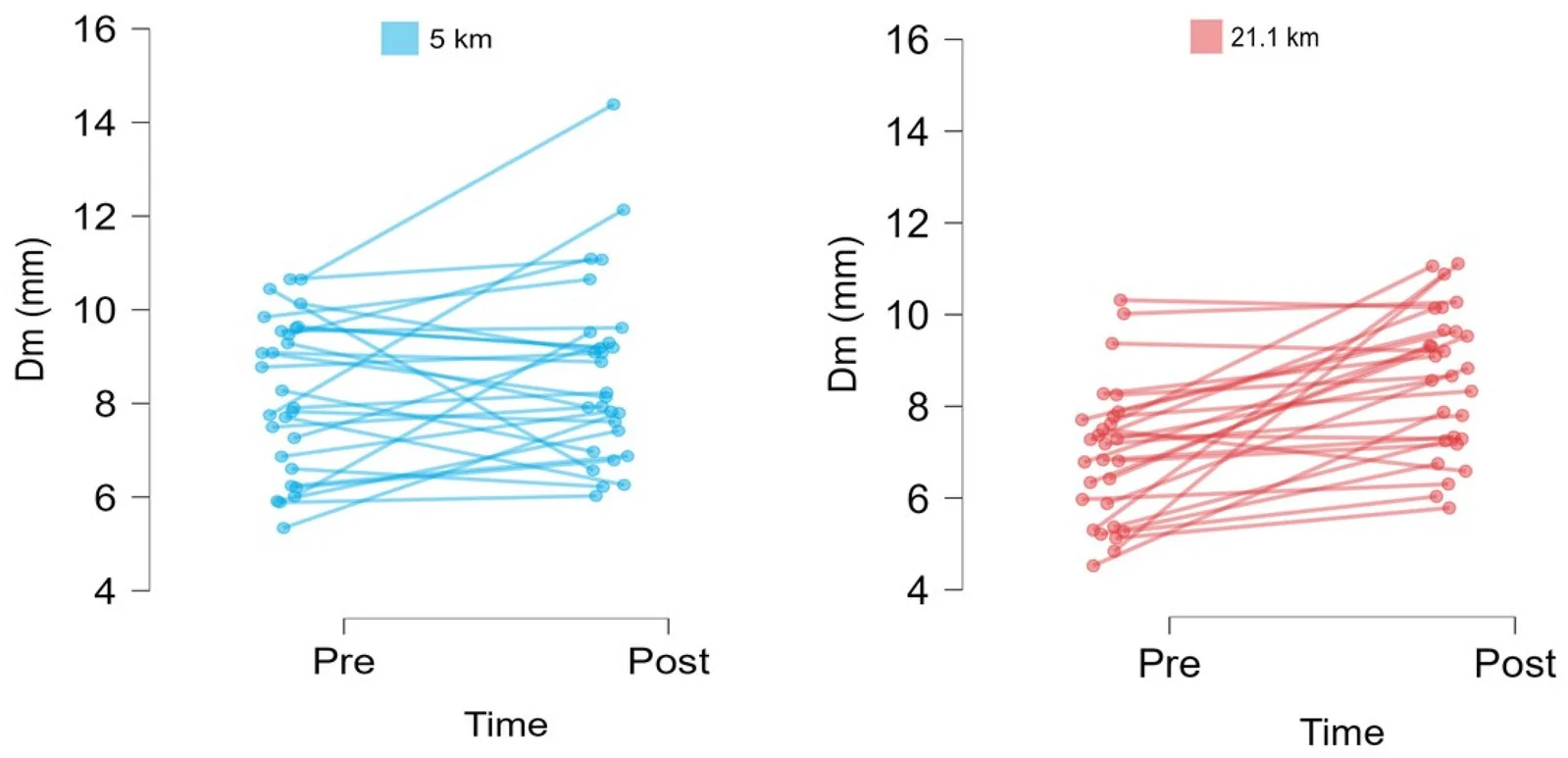
Individual participant trajectories in vastus medialis maximal radial displacement (Dm) before and after the 5 km and half-marathon running trials. Note. The left panel shows the 5 km trial and the right panel the half-marathon trial. Individual lines represent participant-level Dm values averaged across the right and left legs.

**Table 1:** Participant characteristics, running performance, and cardiovascular response during the 5 km and half-marathon running trials.

Variable	M ± SD	Min–Max
**Participant characteristics**		
Age (years)	36.96 ± 11.35	19–59
BMI (kg/m^2^)	23.81 ± 2.40	19.7–31.7
Muscle mass (%)	39.25 ± 3.43	31.9–44.5
Body fat (%)	18.91 ± 5.44	8.7–32.1
**Running indicators**		
5 km pace (s/km)	245.37 ± 35.25	181–310.6
21.1 km pace (s/km)	275.65 ± 32.06	206.2–322.7
5 km relative performance (%)	94.90 ± 3.39	84.62–98.84
21.1 km relative performance (%)	94.36 ± 3.93	84.31–99.96
5 km mean HR (bpm)	164.46 ± 14.94	131–198
21.1 km mean HR (bpm)	156.50 ± 13.62	127–188
5 km peak HR (bpm)	180.21 ± 16.09	142–210
21.1 km peak HR (bpm)	172.75 ± 15.41	139–202

Note. BMI = body mass index; HR = heart rate; bpm = beats per minute. Relative performance was calculated as the best completion time reported for the corresponding distance during the preceding 12 months divided by the trial completion time × 100.

**Table 2:** Tensiomyographic and isometric dynamometry outcomes before and after the 5 km and half-marathon running trials.

Variable	5 km Pre (M ± SD)	5 km Post (M ± SD)	21.1 km Pre (M ± SD)	21.1 km Post (M ± SD)
**TMG parameters**				
VM Dm R (mm)	8.04 ± 2.22	8.47 ± 2.32	6.72 ± 2.06	8.06 ± 1.96
VM Dm L (mm)	8.35 ± 1.69	8.80 ± 2.72	7.18 ± 1.45	9.08 ± 1.55
VM Tc R (ms)	25.24 ± 3.67	24.36 ± 4.45	25.25 ± 4.36	23.71 ± 3.46
VM Tc L (ms)	24.41 ± 3.96	22.76 ± 2.53	24.39 ± 4.21	23.60 ± 2.76
VM Vc R (mm/s)	256.40 ± 69.43	290.90 ± 122.08	219.60 ± 75.82	276.00 ± 71.89
VM Vc L (mm/s)	280.90 ± 68.69	314.00 ± 104.90	242.90 ± 63.06	314.80 ± 77.06
TMG asymmetry (%)	14.57 ± 6.98	15.68 ± 8.46	14.39 ± 6.53	11.32 ± 4.82
**MVIC parameters**				
MVIC R (N)	308.59 ± 103.08	261.56 ± 91.13	311.76 ± 109.91	276.73 ± 118.58
MVIC L (N)	269.61 ± 90.35	247.67 ± 78.57	278.56 ± 104.74	242.02 ± 90.17
MVIC asymmetry (%)	18.32 ± 12.22	14.92 ± 8.40	16.65 ± 11.59	15.53 ± 14.43

Note. VM = vastus medialis; Dm = maximal radial muscle displacement; Tc = contraction time; Vc = contraction velocity between 10% and 90% of Dm; TMG = tensiomyography; MVIC = maximal voluntary isometric contraction; R = right leg; L = left leg; Pre = before running; Post = after running. TMG and MVIC asymmetry values represent absolute interlimb asymmetry, with higher values indicating greater between-leg differences.

**Table 3:** Repeated-measures ANOVA results for TMG parameters and quadriceps MVIC.

Effect	Dm	Tc	Vc	MVIC
**Running trial**	F = 5.862, *p* = 0.022, ηp^2^ = 0.178	F = 0.009, *p* = 0.926, ηp^2^ < 0.001	F = 4.179, *p* = 0.051, ηp^2^ = 0.134	F = 0.249, *p* = 0.622, ηp^2^ = 0.009
**Time**	F = 23.062, *p* < 0.001, ηp^2^ = 0.461	F = 7.187, *p* = 0.012, ηp^2^ = 0.210	F = 27.991, *p* < 0.001, ηp^2^ = 0.509	F = 24.563, *p* < 0.001, ηp^2^ = 0.476
**Leg**	F = 3.387, *p* = 0.077, ηp^2^ = 0.111	F = 4.815, *p* = 0.037, ηp^2^ = 0.151	F = 7.596, *p* = 0.010, ηp^2^ = 0.220	F = 12.075, *p* = 0.002, ηp^2^ = 0.309
**Running trial × Time**	F = 5.756, *p* = 0.024, ηp^2^ = 0.176	F = 0.013, *p* = 0.911, ηp^2^ < 0.001	F = 2.180, *p* = 0.151, ηp^2^ = 0.075	F = 0.009, *p* = 0.925, ηp^2^ < 0.001
**Running trial × Leg**	F = 1.053, *p* = 0.314, ηp^2^ = 0.038	F = 1.340, *p* = 0.257, ηp^2^ = 0.047	F = 0.224, *p* = 0.640, ηp^2^ = 0.008	F = 0.642, *p* = 0.430, ηp^2^ = 0.023
**Time × Leg**	F = 0.465, *p* = 0.501, ηp^2^ = 0.017	F < 0.001, *p* = 0.992, ηp^2^ < 0.001	F = 0.210, *p* = 0.651, ηp^2^ = 0.008	F = 1.161, *p* = 0.291, ηp^2^ = 0.041
**Running trial × Time × Leg**	F = 0.651, *p* = 0.427, ηp^2^ = 0.024	F = 2.468, *p* = 0.128, ηp^2^ = 0.084	F = 0.477, *p* = 0.496, ηp^2^ = 0.017	F = 2.435, *p* = 0.130, ηp^2^ = 0.083

Note. Dm = maximal radial muscle displacement; Tc = contraction time; Vc = contraction velocity; MVIC = maximal voluntary isometric contraction. All effects were tested with df = 1, 27.

**Table 4:** Repeated-measures ANOVA results for absolute interlimb asymmetry.

Effect	TMG Interlimb Asymmetry	MVIC Interlimb Asymmetry
**Running trial**	F = 3.699, *p* = 0.065, ηp^2^ = 0.120	F = 0.067, *p* = 0.798, ηp^2^ = 0.002
**Time**	F = 0.516, *p* = 0.479, ηp^2^ = 0.019	F = 1.253, *p* = 0.273, ηp^2^ = 0.044
**Running trial × Time**	F = 2.970, *p* = 0.096, ηp^2^ = 0.099	F = 0.284, *p* = 0.598, ηp^2^ = 0.010

Note. All effects were tested with df = 1, 27. Higher values indicate greater absolute interlimb asymmetry.

**Table 5:** Descriptive analysis of directional interlimb asymmetry and participant-level changes in asymmetry direction.

**Panel A. Directional Interlimb Asymmetry**				
**Variable**	**5 km Pre**	**5 km Post**	**21.1 km Pre**	**21.1 km Post**
**MVIC directional asymmetry M ± SD (%)**	11.77 ± 18.80	4.05 ± 16.87	9.16 ± 18.30	9.23 ± 19.24
MVIC: right > left, n (%)	24 (85.7)	16 (57.1)	21 (75.0)	19 (67.9)
MVIC: left > right, n (%)	4 (14.3)	12 (42.9)	7 (25.0)	9 (32.1)
**Dm directional asymmetry M ± SD (%)**	−4.05 ± 23.77	−2.29 ± 28.69	−7.25 ± 24.39	−11.02 ± 18.21
Dm: right > left, n (%)	12 (42.9)	14 (50.0)	10 (35.7)	6 (21.4)
Dm: left > right, n (%)	16 (57.1)	14 (50.0)	16 (57.1)	20 (71.4)
**Tc directional asymmetry** **M ± SD (%)**	3.25 ± 13.92	5.33 ± 17.38	3.15 ± 11.55	0.12 ± 9.51
Tc: right > left, n (%)	17 (60.7)	20 (71.4)	15 (53.6)	14 (50.0)
Tc: left > right, n (%)	11 (39.3)	8 (28.6)	11 (39.3)	12 (42.9)
**Panel B. Participants who changed the side with the higher value from Pre to Post**				
**Variable**	**5 km, n/N (%)**		**21.1 km, n/N (%)**	
Dm	10/28 (35.7)		8/24 (33.3)	
Tc	11/28 (39.3)		9/25 (36.0)	
MVIC	12/28 (42.9)		8/28 (28.6)	

Note. Positive values indicate higher values in the right leg and negative values higher values in the left leg. Percentages in Panel A were calculated relative to the full sample (N = 28). Directional asymmetry was examined descriptively. For Panel B, participants with equal right- and left-leg values at Pre or Post were excluded from the corresponding denominator.

## Data Availability

The data supporting the findings of this study are available from the corresponding author upon reasonable request.

## Supplementary Materials

The following supporting information can be downloaded at: https://www.mdpi.com/article/10.3390/biology15191682/s1, Table S1. Normality diagnostics for absolute interlimb asymmetry variables; Figure S1. Distributional plots for TMG-derived absolute interlimb asymmetry; Figure S2. Distributional plots for MVIC-derived absolute interlimb asymmetry; Table S2. Wilcoxon signed-rank sensitivity analyses for absolute interlimb asymmetry; Table S3. Baseline-adjusted linear mixed-effects model for post-running Dm.
