# Supplementary material for: Acute Neuromuscular Responses and Interlimb Asymmetry Following 5 km and Half-Marathon Running in Recreational Runners
Source: Biology (Basel). 2026 Sep 22;15(19):1682. doi: 10.3390/biology15191682 (PMC13663396; doi:10.3390/biology15191682)
Supplement: Supplementary file 1 [file biology-15-01682-s001.zip › biology-4482924-supplementary.pdf]

Table S1. Normality diagnostics for absolute interlimb asymmetry variables

Variable	Skew	Kurt	S-W	p
TMG asymmetry – 5 km Pre	.738	.628	.961	.368
TMG asymmetry – 5 km Post	.871	−.030	.912	.022
TMG asymmetry – 21.1 km Pre	.518	−.148	.965	.462
TMG asymmetry – 21.1 km Post	.404	−.035	.974	.697
MVIC asymmetry – 5 km Pre	.409	−1.131	.906	.015
MVIC asymmetry – 5 km Post	.710	−.494	.926	.049
MVIC asymmetry – 21.1 km Pre	.717	−.653	.906	.016
MVIC asymmetry – 21.1 km Post	1.155	.674	.876	.003

Note. TMG = tensiomyography; MVIC = maximal voluntary isometric contraction; S-W = Shapiro–Wilk statistic; $N = 28$ for all variables. Kurtosis values represent excess kurtosis. The standard errors were .441 for skewness and .858 for kurtosis. Values of $p < .05$ indicate a significant deviation from normality according to the Shapiro–Wilk test.

Figure S1. Distributional plots for TMG-derived absolute interlimb asymmetry.

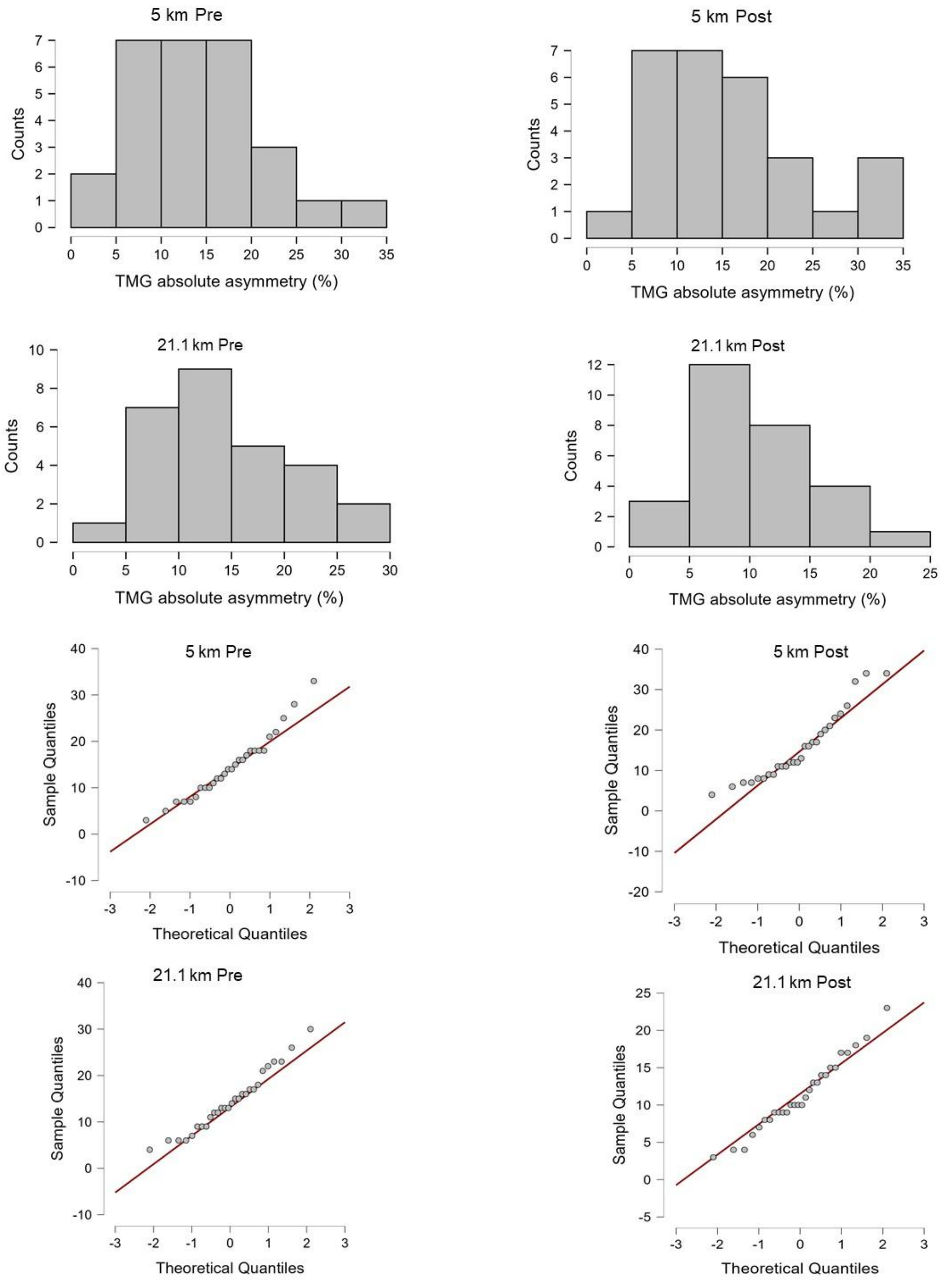

Figure S2. Distributional plots for MVIC-derived absolute interlimb asymmetry.

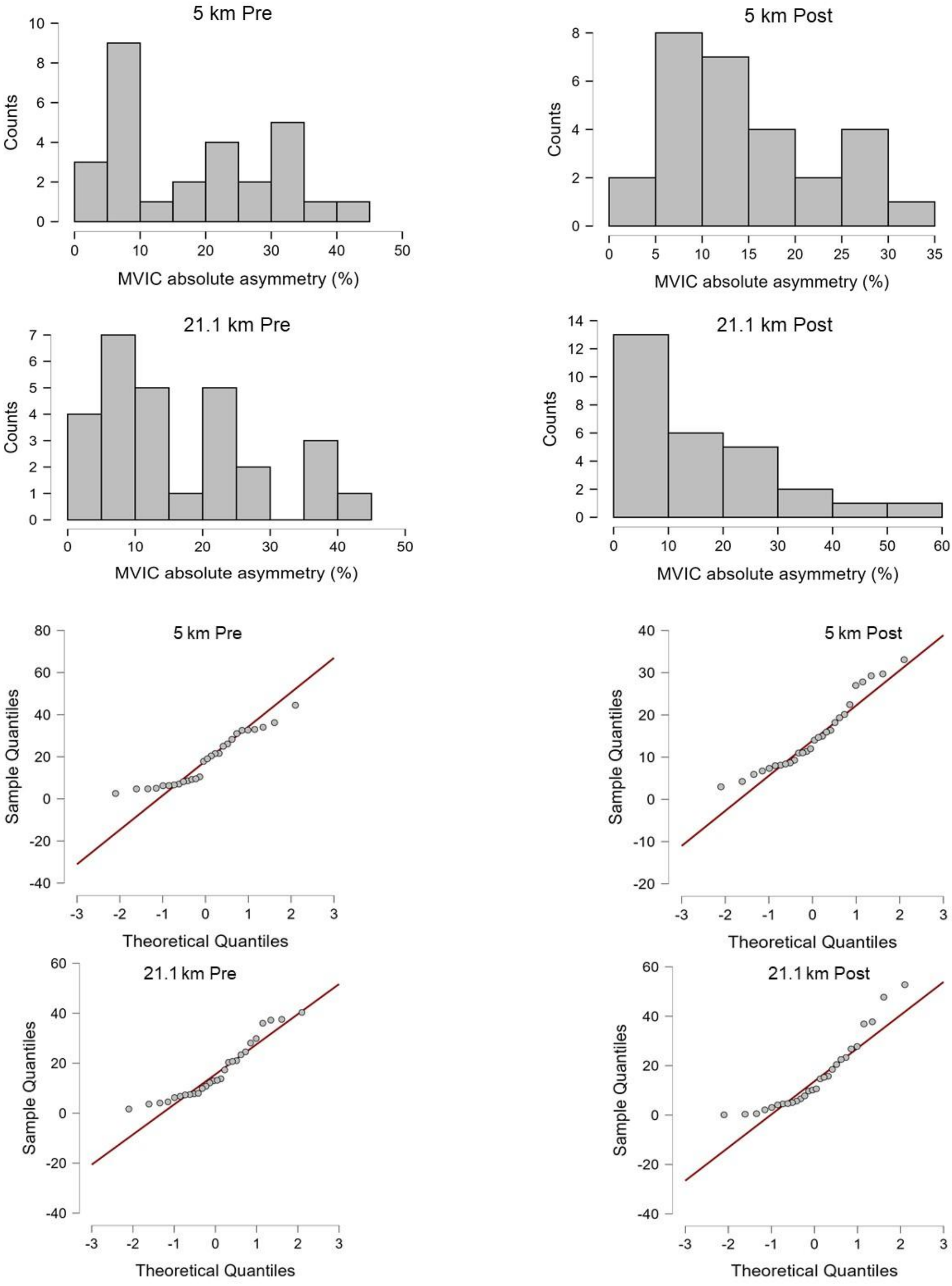

Table S2. Wilcoxon signed-rank sensitivity analyses for absolute interlimb asymmetry

Outcome	Contrast represented	W	p	r_rb
TMG absolute asymmetry	Time: mean Pre vs mean Post	229.5	.553	.131
MVIC absolute asymmetry	Time: mean Pre vs mean Post	264.0	.171	.300
TMG absolute asymmetry	Running trial: mean 5 km vs mean 21.1 km	255.0	.115	.349
MVIC absolute asymmetry	Running trial: mean 5 km vs mean 21.1 km	206.0	.955	.015
TMG absolute asymmetry	Trial × Time: 5-km change vs 21.1-km change	254.5	.118	.347
MVIC absolute asymmetry	Trial × Time: 5-km change vs 21.1-km change	184.0	.678	−.094

Note. W = Wilcoxon signed-rank statistic; r_rb = rank-biserial correlation. Nonparametric sensitivity analyses were performed because several absolute asymmetry distributions deviated from normality. The results were consistent with the corresponding repeated-measures ANOVA findings.

Table S3. Baseline-adjusted linear mixed-effects model for post-running Dm

Fixed effect	F (df1, df2)	p
Baseline Dm	15.411 (1, 100.60)	< .001
Running trial	1.415 (1, 85.17)	.238
Leg	2.070 (1, 78.75)	.154
Running trial × Leg	0.729 (1, 78.03)	.396

Note. Dm = maximal radial muscle displacement; df1 = numerator degrees of freedom; df2 = denominator degrees of freedom. Post-running Dm was modeled with baseline Dm as a covariate, running trial and leg as fixed within-subject factors, and participant as a random intercept. Denominator degrees of freedom were estimated using the Satterthwaite method.
